# Effective delivery of Complex Innovative Design (CID) cancer trials—A consensus statement

**DOI:** 10.1038/s41416-019-0653-9

**Published:** 2020-01-06

**Authors:** Sarah P. Blagden, Lucinda Billingham, Louise C. Brown, Sean W. Buckland, Alison M. Cooper, Stephanie Ellis, Wendy Fisher, Helen Hughes, Debbie A. Keatley, Francois M. Maignen, Alex Morozov, Will Navaie, Sarah Pearson, Abeer Shaaban, Kirsty Wydenbach, Pamela R. Kearns, on behalf of the Experimental Cancer Medicine Centres (ECMC) CID trials working group, Christiane Abouzeid, Rubina Ahmed, Sue Bailey, Catherine Blewett, Helen Campbell, Maria Antonietta Cerone, Glen Clack, Natalie Cook, Serban Ghiorghiu, Sarah Halford, Andrew Johnston, Rick Kaplan, Anna Lawson, Emma Lowe, Jacqueline Mathews, Ilaria Mirabile, Leeza Osipenko, Dipak Patel, Claire Potter, Aoife Regan, Marivic Ricamara, Carly Ringrose, Joanne Rodger, Gurcharan K. Sandhu, Francesca Schiavone, Julie Silvester, Matthew R. Sydes, Charles Weller, Angeliki Yiangou

**Affiliations:** 1 Department of Oncology, University of Oxford, Oxford, UK; 2 Cancer Research UK Clinical Trials Unit, Institute of Cancer and Genomic Studies, University of Birmingham, Birmingham, UK; 3 Medical Research Council (MRC) Clinical Trials Unit, University College London, London, UK; 4 Pfizer, Surrey, UK; 5 The Association of the British Pharmaceutical Industry (ABPI), London, UK; 6 Health Research Authority, London, UK; 7 Wendy Fisher Consulting (WFC) Ltd, London, UK; 8 Cardiff and Vale University Health Board, Cardiff, UK; 9 Independent Cancer Patients’ Voice, National Cancer Research Institute (NCRI), London, UK; 10 National Institute for Health and Care Excellence (NICE), London, UK; 11 Pfizer, New York, USA; 12 Oncology Clinical Trials Office, University of Oxford, Oxford, UK; 13 Queen Elizabeth Hospital Birmingham and the University of Birmingham, Birmingham, UK; 14 Medicines and Healthcare products Regulatory Agency (MHRA), London, UK; 15 National Institute for Health Research (NIHR) Birmingham Biomedical Research Centre, Institute of Cancer and Genomic Studies, University of Birmingham, Birmingham, UK; 16 BioIndustry Association (BIA), London, UK; 17 Cancer Research UK, London, UK; 18 Bristol-Myers Squibb Pharmaceuticals Ltd, Uxbridge, UK; 19 Department of Health and Social Care Research, London, UK; 20 University of Sheffield, Sheffield, UK; 21 University of Manchester, Manchester, UK; 22 AstraZeneca, Cambridge, UK; 23 Newcastle University, Newcastle upon Tyne Hospitals NHS Foundation Trust, Newcastle upon Tyne, UK; 24 MRC Clinical Trials Unit, University College London, London, UK; 25 Cancer Research UK Clinical Trials Unit, University of Birmingham, Birmingham, UK; 26 NIHR Clinical Research Network, London, UK; 27 Experimental Cancer Medicine Centres, London, UK; 28 Sheffield Teaching Hospitals NHS Foundation Trust, Sheffield, UK; 29 UCLH Clinical Research Facility, London, UK; 30 University Hospital Southampton NHS Foundation Trust, Southampton, UK; 31 NHS Research Scotland, Clydebank, UK

**Keywords:** Drug development, Adaptive clinical trial

## Abstract

The traditional cancer drug development pathway is increasingly being superseded by trials that address multiple clinical questions. These are collectively termed Complex Innovative Design (CID) trials. CID trials not only assess the safety and toxicity of novel anticancer medicines but also their efficacy in biomarker-selected patients, specific cancer cohorts or in combination with other agents. They can be adapted to include new cohorts and test additional agents within a single protocol. Whilst CID trials can speed up the traditional route to drug licencing, they can be challenging to design, conduct and interpret. The Experimental Cancer Medicine Centres (ECMC) network, funded by the National Institute for Health Research (NIHR), Cancer Research UK (CRUK) and the Health Boards of Wales, Northern Ireland and Scotland, formed a working group with relevant stakeholders from clinical trials units, the pharmaceutical industry, funding bodies, regulators and patients to identify the main challenges of CID trials. The working group generated ten consensus recommendations. These aim to improve the conduct, quality and acceptability of oncology CID trials in clinical research and, importantly, to expedite the process by which effective treatments can reach cancer patients.

## Background

Cancer is diagnosed in around 18 million people every year worldwide, and 9.6 million die of the disease.^1^ With unhealthy lifestyles and increased longevity, the annual incidence of cancer is set to rise to 29.5 million in 2040. However, for the majority of these cancers, effective treatment remains an unmet medical need.^2^ Recent discoveries in cancer biology and especially immuno-oncology have led to an expansion in the number of new cancer therapies entering clinical development but, frustratingly, the traditional drug development pathway is slow with novel agents taking an average of 12 years to reach clinical practice.^3^ This has generated a “bottleneck” of agents and combinations awaiting clinical evaluation.

To overcome this, the traditional pathway is increasingly being overturned in favour of innovative and efficient trial designs that combine multiple clinical questions within a single study. The term “Complex Innovative Design” (CID) trial here is used to describe them. This includes trials that incorporate several drug development phases (such as seamless Phase 1–2 or Phase 2–3 studies); those with adaptive features (such as using dose–response modelling);^4^ those that evaluate multiple treatments for one indication, one treatment for multiple indications; or those that incorporate multiple treatments and multiple indications within a single “master” protocol.^5,6^ Examples of these trials are shown in Table 1.

**Table 1:** Types of CID trials.

		(i) Evaluation of one experimental treatment (E), common to all cohorts	(ii) Comparative evaluation of multiple experimental treatments (E1, E2, E3, …), common to all cohorts	(iii) Non-comparative evaluation of single (E) or multiple experimental treatments (E1, E2, E3, …), each specific for a biomarker-defined cohort
**Population defined by a single disease**	**No biomarker i.e., single cohort defined by disease**	“Standard” RCT investigating E vs Control (C)	Multi-arm multistage (MAMS) design comparing E1 vs E2 vs E3 etc. vs C e.g., **STAMPEDE**^29^	Not relevant for trials with no biomarker
	**Population stratified by single biomarker (B) i.e., two cohorts: B** **+** **and B−**	Stratified biomarker design investigating E vs C within each cohort	Biomarker stratified MAMS design comparing E1 vs E2 vs E3 etc. vs C separately in B + and B−	Biomarker strategy design evaluating the strategy of using E in B + cohort and C in B− cohort
	**Population stratified by multiple biomarkers** **i.e., biomarkers B1** **+** **, B2** **+** **, B3** **+** **… and B-**	Stratified biomarker design investigating E vs C within each cohort	Biomarker stratified MAMS design comparing E1 vs E2 vs E3 etc. vs C within each biomarker cohort e.g., **I-SPY2**,^30^ **BATTLE**^31^	Umbrella trial evaluating treatment E1 in B1 + cohort, E2 in B2 + cohort etc. Experimental treatments could be allocated to a basket of biomarker cohorts e.g., **PROFILE 1001**,^14^ **FOCUS4**,^32^ **National Lung Matrix Trial**^21^
**Population defined by multiple diseases**	**No biomarker i.e., multiple cohorts defined only by disease**	Stratified RCT investigating E vs C across a basket of disease types	Stratified MAMS design comparing E1 vs E2 vs E3 etc. vs C across a basket of disease types	Not relevant for trials without biomarkers
	**Population stratified by single biomarker i.e., two cohorts B** **+** **and B- within each disease**	Stratified biomarker design investigating E vs C within each biomarker cohort across a basket of disease types	Stratified MAMS design comparing E1 vs E2 vs E3 etc. vs C separately in B + and B− across a basket of disease types	Biomarker strategy design evaluating the strategy of using E in B + cohort and C in B− cohort across a basket of disease types
	**Population stratified by multiple biomarkers i.e., biomarkers B1** **+** **, B2** **+** **, B3** **+** **, … and B- within each disease**	Stratified biomarker design investigating E vs C within each biomarker cohort across a basket of disease types	Stratified MAMS design comparing E1 vs E2 vs E3 etc. vs C within each biomarker cohort across a basket of disease types	Umbrella trial evaluating treatment E1 in B1 + cohort, E2 in B2 + cohort etc. across a basket of disease types e.g., **NCI-MATCH**^33^

(i) Descriptions specify the comparison of experimental arms to a control arm C, but designs can include experimental arms that would be compared to historical controls; (ii) all designs described that include multiple cohorts or multiple experimental treatments can also be dynamic platform trials; (iii) all designs can be adaptive and incorporate seamless transition from one phase to the next

Examples of studies shown in Bold type

So far, the main CID trials to have been conducted are “master protocol” trials that incorporate molecular biomarkers to define patient cohorts. These include “basket” and “umbrella’ designs”.^7^ Unlike conventional clinical trials in which patients are recruited by their tumour of origin, patients enrolled in basket trials have different tumour types, but all have a common molecular characteristic (a biomarker) relevant to the treatment under investigation. By contrast, in umbrella trials, patients with a single-tumour type are stratified into multiple cohorts based on molecular markers defining each treatment arm. These stratifications allow parallel comparison of therapy/ies for an individual disease (or biomarker cohort) or enable overall assessment via a single stratified analysis. In addition, treatments and patient cohorts can be added or discontinued whilst the trial is ongoing.

CID trials have long been recognised by regulators and other agencies as important tools in drug development. In 2007, the European Medicines Agency (EMA) provided guidance on the introduction of adaptive measures in trials and, in 2011, on risk-based quality management.^8,9^ Within their 2017 Life Sciences Industrial Strategy, the UK Government committed investment towards clinical trials that incorporate “novel methodology” and their subsequent Sector Deal 2 was agreed, in partnership with industry, to fund the research infrastructure to deliver them.^10,11^ Outside Europe, the US Food and Drug Administration (FDA) released draft guidance around risk-based monitoring in 2013 and around master protocols and adaptive designs in 2018. The FDA have also launched a pilot programme to support CID trials that accelerate drug development in areas of unmet need.^12,13^

One of the earliest examples of a CID trial was the international PROFILE 1001 non-small cell lung cancer (NSCLC) study designed to investigate crizotinib, a targeted inhibitor of the tyrosine kinase cMET.^14^ As data emerged that rearrangements of the Anaplastic Lymphoma Receptor Tyrosine kinase (ALK) gene were present in a small subset of NSCLC patients, new molecularly defined study cohorts were added to capture this population. As a result, EU Marketing Authorisation was obtained for crizotinib just 5 years after the discovery of the *EML4-ALK* fusion gene.^15,16^ Examples of other CID trials are shown in Table 1.

## Aims

Drawing on the experiences of multiple stakeholders involved in CID trials, we described the pathway and hurdles encountered when conducting these trials and formulated consensus recommendations to navigate them. Whilst we used the UK clinical research infrastructure as a test case, the experiences are applicable to all international stakeholders involved in CID trials and highlight their increasing importance in both academic and commercial development. Although the focus of this review is oncology based, its recommendations are applicable to other disease areas.

### Method: evaluating the landscape and formulating the consensus recommendations

**Fig. 1:**
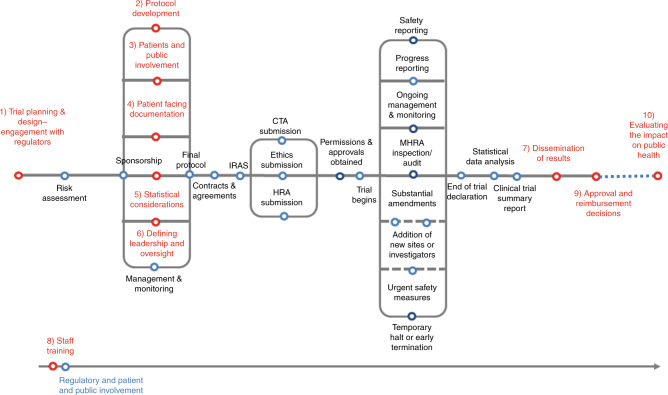
CID Trial Pathway (adapted from the NIHR Clinical Trials Toolkit Routemap^28^) showing the stages of clinical trial development leading to licensing and approval (blue). Stages shown in red correspond to Consensus Recommendations defined in this paper that are of particular relevance to CID trials.

The UK hosts a networked infrastructure of adult and paediatric early-phase trial centres; the Experimental Cancer Medicine Centres (ECMC) network.^17^ Each centre exists as a partnership between National Health Service (NHS) Trusts, Health Boards and Universities in order to facilitate translational (or “bench-to-bedside”) research. As well as conducting early-phase studies, ECMC representatives advise on research conduct and policy and contribute to Department of Health and Social Care (DHSC) and National Institute of Health Research (NIHR) steering and advisory committees. At the ECMC Annual Network meeting in 2017 it became clear that, although stakeholders acknowledged that CID trials could accelerate drug development, few had expertise in conducting them. Workshops were held in February and June 2018 with relevant stakeholders from academia, funding bodies, regulators, health technology assessment (HTA) and industry including representatives from The Association of the British Pharmaceutical Industry (ABPI), BioIndustry Association (BIA), Cancer Research UK (CRUK), DHSC, Cardiff and Vale University Health Board, NHS Greater Glasgow and Clyde, Health Research Authority (HRA), Medicines and Healthcare products Regulatory Agency (MHRA), Clinical Trial Units (CTUs), Research Ethics Committees (RECs), academic institutions, National Institute of Health and Care Excellence (NICE), National Institute of Health Research (NIHR), patients, Independent Cancer Patient Voices (ICPV), researchers and NHS Trust Research and Development (R&D) Managers from across the UK (the ECMC CID working group). A writing workshop focused upon the most challenging aspects of CID trials in order to provide recommendations on how they could be addressed. While it was agreed that most aspects of designing and conducting CID trials are similar to those of any clinical trial, CID trials present unique challenges at specific points along the clinical trial pathway (see Fig. 1). A summary of the ten consensus recommendations are provided in Table 2.

**Table 2:** Summary of consensus recommendations.

*Recommendation 1—Trial Planning and Design – Engagement with Regulators* Investigators/sponsors should arrange a joint meeting with regulators, HTA bodies and other key stakeholders as early as possible before or during the trial design to guide and shape the delivery of the CID trial, especially if accelerated (e.g., conditional) approval is likely to be applied for.
*Recommendation 2—Protocol Development* The protocol should identify and briefly describe any possible future modifications (such as additional study arms) to reduce the likelihood of substantial amendments. Events defining the end of trial must be included.
*Recommendation 3—Patients and public involvement (PPI)* Patients and the public may require specific training, support, and perhaps also accreditation, in order to review and/or manage CID trials.
*Recommendation 4—Patient Facing Documentation* A practical approach using three-part patient information should be provided; comprising an invitation document, a study arm-specific document and a handbook. Multimedia can be considered for some or all of these documents.
*Recommendation 5 —Statistical Considerations* Experienced and detailed statistical input is required to provide an overarching statistical design with flexibility to incorporate individual variations for different treatments, diseases and molecular characteristics. Having a range of expertise within the oversight committee will ensure timely and appropriate responses to the frequent analyses produced.
*Recommendation 6—Defining Leadership and Oversight* A Trial Management Group with experience of CID trials should be convened to oversee the study. New Chief Investigators (CIs) and/or Principal Investigators (PIs) should be appointed during the study as its requirements evolve.
*Recommendation 7—Dissemination of Results* When a research question is answered, or a study arm is completed, timely reporting of trial data at these pre-specified time points should be supported as best practice.
*Recommendation 8 —Staff Training* Training in complex trial methodologies should be included in the undergraduate and post-graduate training curricula of relevant health care professionals in order to ensure appropriate resources are in place to deliver CID trials.
*Recommendation 9 —Approval and Reimbursement Decisions* Accelerated-access initiatives are vital in ensuring CID trial findings are rapidly transitioned to regulatory approval, reimbursement decisions and adoption into clinical practice.
*Recommendation 10 —Evaluating the impact on public health* Impact analyses should be conducted on all CID trials to ensure they deliver on their promise to provide timely access to these medicines in clinical practice without compromising patient safety.

## Consensus recommendations

### Trial planning and design—early stakeholder engagement

#### Recommendation 1: trial planning and design—engagement with regulators

Investigators/sponsors should arrange a joint meeting with regulators, HTA bodies and other key stakeholders as early as possible before or during the trial design to guide and shape the delivery of the CID trial, especially if accelerated (e.g., conditional) approval is likely to be applied for.

As with any clinical trial involving an Investigational Medicinal Product (IMP), approval of a clinical trial authorisation (CTA) application is required before a CID trial can be conducted. In Europe, this is obtained from a regulatory “Competent Authority”, for example, the MHRA in the UK or the HPRA in Ireland. To maximise the chance of a successful application, sponsors of CID trials should arrange an advice meeting at an early-stage of protocol development with both the Competent Authority and other interested parties, such as health technology assessment (HTA) bodies, NICE or SMC. Commercial sponsors may also seek advice from the EMA and EUNetHTA. A joint meeting of this type has the advantage of ensuring that the objectives and design of the study meet the requirements of all parties (i.e., demonstrate a positive risk/benefit balance for the IMP as well as its clinical and cost-effectiveness) and are appropriate to support CTA approval as well as future marketing authorisation and reimbursement decisions. At these meetings, the overarching trial aims and specific aspects of the study protocol or development programme, the selection of the patient population, the choice of comparator and the endpoints can be addressed.

These discussions are particularly relevant for IMPs that may be destined for accelerated (e.g., conditional) marketing authorisation. As the accelerated approval pathway is a relatively novel concept in UK (and in the EU), the choice of surrogate endpoints, for example, must be carefully considered. In particular, as to whether the surrogates (for example, rate of pathological complete response) are likely to adequately predict clinical outcomes relevant to NICE and most HTAs in Europe, such as overall survival. This is particularly important if they are to be evaluated later, once marketing authorisation has been obtained. Of note, basket trials should be comparative and randomised whenever possible to support reimbursement decisions with the choice of active comparator ideally reflecting clinical practice. Non-comparative basket studies (consisting of several single arm studies) should only be conducted when comparative trials are not feasible, and a justification of this design should be documented.

Even when expedited approval is not anticipated, the overarching aim of these advice meetings is to gain mutual understanding on how the study design will enable the trial hypothesis to be tested effectively and thereby help towards securing trial and marketing approval. By working together in this way, regulators, HTA, sponsors and investigators can refine their knowledge and understanding of how to safely conduct successful CID trials (for examples of meeting options in the UK see Table 3). It is important that any learning and best practice acquired are then shared between relevant regulatory authorities and academic communities in the UK, EU and US to further support the body of evidence around this type of clinical research.

**Table 3:** UK options for obtaining advice.

**Type of advice**	**Scope**
MHRA Regulatory scientific advice https://www.gov.uk/guidance/medicines-get-scientific-advice-from-mhra	The questions should address specific scientific issues on quality, non-clinical, clinical or regulatory aspects.
MHRA Broader Scope advice https://www.gov.uk/guidance/medicines-get-scientific-advice-from-mhra	A broader scope meeting is not a product-specific request, but a broader discussion. Examples include: • practical issues of study design, management and analysis • general approaches to product development • overall product development plans where there are very broad issues that may go beyond what can be discussed at a routine scientific advice meeting
NICE scientific advice (and parallel advice with the MHRA): https://www.nice.org.uk/about/what-we-do/life-sciences/scientific-advice Contact: https://www.snapsurveys.com/wh/s.asp?k=152629077520	NICE offers fee-based consultancy service to developers of pharmaceuticals or biopharmaceuticals. NICE scientific advice helps develop evidence that demonstrates the value of investigational medicinal products (IMP). NICE provide detailed advice on clinical (population, interventions, comparators and outcomes), economic and evidence generation plans, help to integrate cost-effectiveness considerations into evidence generation plans.
MHRA scientific advice (and parallel advice with NICE): https://www.gov.uk/guidance/medicines-get-scientific-advice-from-mhra	At these meetings clinical trials sponsors will be able to discuss clinical study design that can satisfy regulatory and NICE requirements. Sponsors can also get optional input from the Clinical Practice Research Datalink (CPRD).
MHRA regulatory advice CTU.AdviceMeetings@mhra.gov.uk	This service provides information, advice and guidance that clarifies UK and EU regulatory requirements. This service is often, but not exclusively, utilised by academic Sponsors.
MHRA Innovation Office https://www.gov.uk/government/groups/mhra-innovation-office	The MHRA Innovation Office is open to queries about innovation in medicines, medical devices and novel manufacturing processes—particularly those that challenge the current regulatory framework. The office provides access to world-class knowledge, expertise and experience from specialists across MHRA (including the Inspectorate, Enforcement and Standards Unit), CPRD (Clinical Practice Research Datalink) and NIBSC (National Institute for Biological Standards and Controls). Experts from the Health Research Authority and Human Tissue Authority can also be requested.
MHRA CTU Help line clintrialhelpline@mhra.gov.uk	It is possible to contact the CTU directly for specific queries about clinical trials or aspects that do not require an advice meeting.
HRA Queries HRA.Queries@nhs.net	General queries on the health research process, including research ethics.
IRAS http://www.myresearchproject.org.uk/help	Application portal for research approvals (MHRA, HRA, REC)
NHS Research Scotland http://www.nhsresearchscotland.org.uk/ enquiries@nrs.org.uk	Support in Scotland

### Protocol development

#### Recommendation 2: protocol development

The protocol should identify and briefly describe any possible future modifications (such as additional study arms) to reduce the likelihood of major amendments. Events defining the end of trial must be included.

#### Structuring a protocol

CID trials can be presented as a single trial with a single protocol (e.g., core/master protocol plus individual parts/modules/appendices) or as separate trials, with the master/core protocol submitted each time, but with additional modules. It is essential that all parts of a CID protocol are clearly worded to enable easy navigation between them. Although there is no standardised CID protocol, modifiable templates are available in the UK from the HRA.^18^

#### Defining the trial hypothesis and primary objectives

A CTA application is more likely to be successful if the overarching trial hypothesis is clearly defined and the primary study objective(s) are designed to answer it in a specific, timely and scientifically valid way. This will enable clarification of when an amendment or a new trial is required to address an incoming research question and reduce the likelihood of generating results that are insufficient to meet regulatory requirements.

#### Identifying adaptations

Due to the duration and complexity of CID trials, it is likely that amendments to its design or delivery will be required during its course. As amendments are costly and time-consuming, they can, to some extent, be mitigated by defining clear parameters in the initial application, such as anticipated future modifications. These include, for example, stating which future patient populations are likely be evaluated, the number of IMPs or future study arms that are likely to be added and the criteria to decide this, how dosage alterations will be decided and how unexpected toxicities might alter the conduct and design of the trial. In addition, a plan should be in place to oversee and use emerging outcome data, to decide which external developments could alter the protocol and how and when (and with what evidence) biomarker selection criteria could change. If details are unknown at the time of the initial application, for example, which future dosing regimens will be selected, all available data should be included with the caveat that a later amendment will be filed to provide more information. A sponsor wishing to add new arms or IMPs to an ongoing trial should seek advice from the regulators prior to submitting an amendment to determine if it is considered substantial or non-substantial; if this modification was predicted in the original protocol, it is more likely to be defined as being non-substantial.

Appropriate resource should be available for both central coordination and study site/per patient costs in CID trials. Over time, changes in the approved use of agents and standard of care agents can occur, so consideration should be given to how such changes will be managed during the life of the trial and the costing model should reflect this. Upfront costs should adequately meet the administrative burden to set up CID studies, as well as to manage potential multiple amendments and the inclusion of additional study arms.

#### Data and databases

The data in CID trials are often complex, obtained from multiple sources (e.g., clinical, laboratory/biomarker, imaging and safety) and used for decision-making at many points in the study to determine whether particular cohorts will continue. Therefore, the protocol must include details and a clear definition of the minimum data required for such decisions. IT systems will need to be designed, tested and validated to appropriately manage the entry and registration of patients as well as facilitate the collation of data, monitoring and reporting of progress throughout the trial. Flexibility of the system will be important where treatment schedules, cohorts and interventions are likely to change.

#### Contracts

CID trials may require the involvement of multiple parties: funders, study sites, IMP providers, laboratories and third-party service providers. Planning and resourcing for this aspect of set-up should also reflect the number and range of contracts which will be required, as well as the timelines for negotiation and agreement.

#### Risk assessment and monitoring

Risk assessment is particularly important for CID trials, and should include consideration of financial and operational risks alongside the trial management activities required to mitigate them. Early consideration of the level of source data verification (SDV) required should feature in trial planning, in which data will be used for decision points and for regulatory registration packages. Feasibility of the desired SDV levels should be considered too, as some sites may be unable to host the volume or frequency of monitoring that is required. Risk-based monitoring is a data-driven approach which can lead to improvement in the efficiency of monitoring while preserving data quality.^19^ This approach has been endorsed by the EMA, and is particularly suited to CID trials given their complexity.^9^

#### Trial management support

Coordination of such trials requires expert trial and programme management. Trial management is usually designed to meet the requirements of distinct sequential study phases: set-up, recruitment, patient follow-up and subsequent analysis and reporting. However, CID trials may have multiple cohorts at different stages open at any given time, complicating the expertise and time required to coordinate activity across multiple phases, protocols/cohorts and with third parties in order to maintain compliance and communication. Additional resource may be required to ensure that communications are adequately managed, input from multiple parties is coordinated and the relevant information and data are made available at the key discussion and decision time points.

#### Defining the end of the trial

Although CID trials are designed to be flexible, a clear trial end needs to be specified, e.g., a maximum study duration, a maximum number of arms or events that define the end of each study arm. This is to prevent the establishment of an endless study, with numerous additional arms or IMP combinations that were not considered in the original trial design. Planning adaptations require a rigorous science peer review, and the review cycle of funding applications needs to be taken into consideration. It is important to discuss with the relevant funding bodies how this review process can be managed efficiently to avoid undue delays for adaptations. In addition, any change to the end of trial, for whatever reason, should be notified as a substantial amendment, with prior regulatory discussions recommended.

### Patient and public involvement (PPI)

#### Recommendation 3: patient and public involvement

Patients and the public may require specific training, support, and perhaps also accreditation, in order to review and/or manage CID trials.

As with all trials, PPI should be embedded in all stages of the process from trial planning and design to dissemination of the results. It is important to ensure the wording of all trial documentation is not unnecessarily technical and is suitable for lay readers. The complicated nature of CID trials and the time commitment required to review the CID trial documents needs to be recognised, and could be met in an accreditation process whereby patients and the public involved in the review and conduct of CID trials are given specific and appropriate training and support.

PPI groups should be invited to review and comment on participant information sheets, to ensure “information overload” is avoided, and the protocol itself to ensure the eligibility criteria are justified and are not unnecessarily discriminatory. This is particularly relevant for protocols in which specific age or performance status cut-offs have been applied or certain comorbidities or past medical conditions are excluded without scientific justification. For disease-specific studies, PPI engagement can provide information about the impact of a disease on the patient’s daily life, from which the appropriate patient reported outcome measures (PROMs) can be selected. Evidence of this involvement should be recorded in trial documentation.

### Patient-facing documentation

#### Recommendation 4: patient-facing documentation

A practical and encouraging approach using three-part patient information should be provided comprising an invitation document, a study arm-specific document and a handbook. Multimedia can be considered for some or all of these documents.

CID trials are invariably complex, and a single patient information sheet is likely to either be too long and complicated, containing non-applicable information, or too brief to be helpful. One suggested approach is for patient-facing documentation for CID trials to be broken down into three documents.

#### Document 1: an invitation document

This should be an introduction to the research as a whole, possibly in the form of an invitation letter outlining the overall objectives of the trial. This should be less than two pages in length. If the patient has yet to be allocated to an arm it should describe how this will be done: i.e., randomisation.

#### Document 2: a study arm-specific document

This should provide the participant with enough information to enable them to understand what taking part will entail specifically for them. It should include a clear study visit schedule, a description of the drugs to be administered, procedures and tests to be conducted, a list of the main risks and any prohibited activities, foods or medications. It should be no more than four pages long, and ideally include a summary flow chart.

#### Document 3: a participant handbook

This should describe the technical and practical details of taking part in the trial, for example, the expenses to be paid, insurance and complaint procedures, legal details such as data protection regulations and information regarding the storage and analyses of tissue biopsies, protection and sharing of the personal data. This document should contain a comprehensive contents page, and be clearly indexed. The handbook should ideally not require amendment as the information contained is unlikely to change during the course of the CID trial. Formats other than the written word should be considered, such as videos and multimedia which have been shown to improve both patient and researcher satisfaction. Within the UK, the HRA and MHRA produced a joint statement in September 2018 endorsing the use of multimedia and eConsent, which removed the requirement for “wet signatures” for regulatory compliance inspection purposes.^20^

### Statistical considerations

#### Recommendation 5: statistical considerations

Experienced and detailed statistical input is required to provide an overarching statistical design with flexibility to incorporate individual variations for different treatments, diseases and molecular characteristics. Having a range of expertise within the oversight committee will ensure timely and appropriate responses to the frequent analyses produced.

Statistical requirements vary by the type of CID trial. In umbrella trials, in which different experimental treatments in different biomarker subgroups within the same protocol are evaluated, an overarching statistical design that is common to all treatment arms can be deployed.^7^ For example, the National Lung Matrix Trial uses a Bayesian adaptive design with a common set of outcome measures, but different primary outcomes and clinically relevant thresholds specified for different biomarker cohorts.^21^ With different biomarkers having different prevalences, rates of recruitment to each cohort can vary dramatically requiring interim analyses at multiple time points. This requires close working with statistical teams and regular updates to oversight committees. Umbrella trials provide a unique opportunity to evaluate treatments for rare biomarker cohorts that would not otherwise be possible, but it may not be feasible to reach a statistically optimal sample size, leaving a greater level of uncertainty.

In basket trials, where a single treatment is being investigated across multiple tumour types, the central statistical approach is to “borrow information” i.e., the analysis of the treatment effect in any individual disease cohort accounts for the effects that are observed in the other parallel cohorts. Various methods have been proposed to do this. The main challenge is determining the level of exchangeability of information across disease types that is biologically valid. CID trials that aim to compare multiple experimental treatments either against each other or a common control arm typically use a multi-arm multistage (MAMS) design allowing interim statistical analyses to drop arms that do not show promise and, if new arms are added within an ongoing platform trial, then statistical comparisons can only be made against contemporaneous patients in other arms.

CID trials are often adaptive, allowing interim trial data to be used to make changes to the design as it proceeds, but such adaptations must be planned upfront and adjusted for within the statistical analysis. The complexity of designs may be more readily addressed by using a Bayesian approach to analyses, which can provide more flexibility and intuitive decision-making than within a hypothesis-testing frequentist framework, but requires complex computer simulations to determine necessary sample sizes and operating characteristics of the design.

The heavier statistical workload to deliver CID trials should not be underestimated when considering the resources required. Such trials often involve more complex statistical methodology and require specialist expertise. Once the trial is running, the availability and responsiveness of the statistical team are critical to the delivery and reporting of the study.

### Trial conduct and delivery considerations—leadership and management of responsibilities and expectations

#### Recommendation 6: defining leadership and oversight

A Trial Management Group with experience of CID trials should be convened to oversee the study. New Chief Investigators (CIs) and/or Principal Investigators (PIs) should be appointed during the study as its requirements evolve.

As with all studies, in CID trials the sponsor is ultimately responsible for the safety of the trial participants and the integrity of the study, but may wish to agree a “level of understanding” between themselves, the investigators and the organisation responsible for conduction of the trial (typically a Clinical Trials Unit (CTU) or a Contract Research Organisation (CRO)) to delegate some responsibilities.

Whilst a conventional trial may answer one primary research question and have a single CI, CID trials present a number of research questions during their life course. Therefore, it may become necessary for CI and PI responsibility to be shared or transferred between specialists over time. This is particularly the case for trials across different cancer types and at different stages of disease where clinicians will have varying levels of sub-specialisation. Trial protocols and contracts must be adaptable to accommodate these leadership changes. This also applies to switching operational and statistical specialists if the design moves from early into later-phase periods of research. The Trial Management Group (TMG) should include members with experience of CID trials, both in terms of scientific design and delivery. The members also need to appreciate that the level of time commitment is likely to be considerably greater than for a conventional trial. The TMG should be prepared to adapt its structure and membership to engage the most suitable expertise at different stages of the programme of work. Moreover, as alterations are introduced to the trial, there may be a need to introduce additional oversight or amend the remit of existing oversight committees, for example, involving Independent Data Monitoring Committees and Trial Steering Committees. An important remit of the Steering Committee is to carefully consider the preclinical and clinical evidence generated around investigational products used in the study alongside that generated from contemporaneous trials using similar products (for example, with the same mechanism of action or sharing similar pharmacodynamic properties). This prevents duplication of futile therapeutic strategies and inefficient use of resources, both being over-riding principles of CID trials.

### Reporting and publishing results

#### Recommendation 7: dissemination of results

When a research question is answered, or a study arm is completed, timely reporting of trial data at pre-specified time points should be supported as best practice.

For many CID trials, the legally defined “end of trial” occurs when all the multiple research questions have been answered, and there are no further data collections or amendments required. However, for a CID trial that is being sequentially adapted by adding or dropping new parallel investigational arms, the primary endpoint in some arms may be reached months or even years before the full trial is completed and the optimal reporting time may be unclear. There may be restrictions on the timing of study reporting, such as to protect the blinding of study data, avoid introducing biases or false discoveries and preserve confidence in the validity of the overall trial results. In addition, many CID trials are conducted under commercial or academic–commercial partnerships with commercial sensitivities to be considered. In some circumstances, full release of data on the biomarker characteristics of responding patients could be of value for the commercial development of an agent. However, to maintain the overall trial integrity, a more restricted interval release of specific outcome data to trial investigators and collaborating companies is required.

Importantly, there is now a legal obligation in the EU and in the US to report both negative and positive clinical trial results either in EudraCT or in clinicaltrials.gov. within a specific time of study closure. Although there is no regulatory mandate to report interim trial data or make the results of completed arms of a CID trial available on public trial registries until the whole trial is completed, the prompt reporting of the results as they are obtained minimises the risk of duplication of effort whilst enabling early dissemination of important findings into the community. It is our recommendation that reporting of trial data at robust and pre-specified time points when scientifically and clinically relevant should be supported as best practice and the most appropriate timing of data release must be predefined in the protocol. It will be crucial to ensure this practice extends beyond scientific publication and includes dissemination to trial participants and other interested groups and communities. In addition, a clear publication policy should be defined at the start of the trial and specified in the protocol. Many CID trials may need to publish under a collective consortium and allowances for this should be made by journals and publishers.

### Evaluating the impact on the trial team and the need for training

#### Recommendation 8: staff training

Training in complex trial methodologies should be included in the undergraduate and post-graduate training curricula for relevant health care professionals in order to ensure appropriate resources are in place to deliver CID trials.

The extended duration of CID trials, the necessity to adapt the trial to emerging data and the inclusion of later-phase and site-specific cohorts means that CID trials require intense involvement from investigators. This requires a high level of PI and trial committee oversight, not only to review and assess their own patients but to participate in regular conference calls/communication with other centres and trial sponsors. They should maintain detailed and up-to-date knowledge of the study accrual across the investigative centres and make decisions based on, often quite preliminary, data. It is important that centres wishing to conduct CID trials recognise the benefit to their patients of these types of trial and ensure there is sufficient time and support for investigators to deliver them safely and that investigators are fully appraised of their roles and responsibilities beforehand.

CID trials provide important opportunities for more junior researchers to learn about the delivery of clinical trials. Training in innovative trial methodologies should be included into the curriculum of medical, nursing and pharmacology students. Once qualified, health professionals should have access to appropriate continued or higher education about clinical trials. Masters courses have already proven successful in training trialists. Conferences with the specific aim of educating doctors in trial conduct also provide an important teaching forum for the next generation as, currently, early-phase trial experience is not mandated within the oncology training curriculum. Furthermore, the UK Government’s Life Sciences Sector Deal 2 has committed to delivering a skills programme to embed expert understanding of how innovative trials can be run across the NHS.^11^ The NIHR Clinical Research Network plans to deliver e-learning courses, designed to increase awareness and understanding of innovative trial designs, supplemented with targeted learning for specific groups.

### Fast-tracking CID trials to the clinic

#### Recommendation 9: approval and reimbursement decision

Accelerated-access initiatives are vital in ensuring CID trial findings are rapidly transitioned to regulatory approval, reimbursement decisions and adoption into clinical practice.

The faster transition of new medicines into clinical practice is not solely achieved by speeding up clinical trials but also requires a conducive legislative environment. In normal circumstances, once a medicine has undergone clinical evaluation, it must receive marketing authorisation and then be appraised by national Health Technology Assessors before becoming available to patients. The “21st Century Cures Act” was passed in the USA to enable the FDA to speed up this pathway for agents showing early clinical promise by implementing priority review, breakthrough therapy, accelerated approval and fast-track approval status.^22^ Sponsors are thus able to seek “breakthrough status” on the basis of surrogate or intermediate clinical endpoints (rather than overall survival) as indicators of clinical promise and apply for fast-track designation. In a similar vein in the EU, following the EMA’s PRIME scheme,^23^ accelerated-access initiatives have been launched to reduce the timeframe to review applications and grant a conditional marketing authorisation. Similarly, the UK government’s Accelerated Access Collaborative (AAC) confers “transformative designation” on drugs, diagnostics, medical and digital technologies so they can enter an Accelerated Access Pathway (AAP).^24^ In 2019, the remit of the AAC was expanded to include all UK health innovation.^25^ By these mechanisms, a conditional marketing authorisation is granted to manufacturers provided they fulfil post-authorisation commitments to address any areas of uncertainty and/or evidence gaps to fully characterise the full risk/benefit profile of their product. Despite the obvious advantages of these rapid-access schemes, the early approval of medicines is not without risk. Some anticancer products that benefited from fast-tracked approval by the FDA on the basis of surrogate endpoints, such as overall response rate or duration of response, subsequently failed to show adequate confirmatory clinical outcomes or demonstrated emergent safety concerns. It is therefore essential that regulators specify the appropriate post-marketing commitments to manufacturers, and that marketing authorisation is withdrawn if these are not met.^26,27^

### Evaluating the public health impact of CID trials

#### Recommendation 10: evaluating the impact on public health

Impact analyses should be conducted on CID trials to ensure they deliver on their promise to provide timely access to these medicines in clinical practice without compromising patient safety.

No formal comparisons of CID with traditional studies (randomised controlled clinical trials) have yet been performed to confirm they do indeed provide a faster route to clinic. Impact analyses are therefore required to fully evaluate the effectiveness, speed and efficiency of CID studies. In addition, ongoing appraisal must be conducted of the cost and public health consequences of fast-tracking medicines to patients to ensure they are clinically advantageous and cost-effective. This also confers greater reliance on post-authorisation and conditional reimbursement data collection (e.g., via the Cancer Drugs Fund) based on clinically relevant clinical endpoints (e.g., overall survival, PROMs) to provide a fully comprehensive assessment of the risks and benefits of fast-tracking the development of cancer medicines.

## Conclusions

By using the ECMC network to bring together stakeholders and share their perspectives, ten consensus recommendations have been developed to provide a framework of multi-stakeholder guidance on conducting CID trials. Early involvement from competent authorities and HTA bodies into the design of these studies can assure their success and rapid adoption. As the concept of CID studies is still relatively novel, it is important that expertise gained from conducting them is shared between regulatory authorities and academic communities within and beyond the UK. Furthermore, formal collaborations should be established between global regulators to disseminate universal best practice in all aspects of these studies and across other therapeutic areas. To optimise the utility of these trials, accelerated approval schemes are in development to fast-track IMPs showing positive early efficacy signals. Although challenging, CID studies have the potential to facilitate wider and faster access to treatment innovation, to enhance the therapeutic options available to cancer patients and to accelerate the development of treatments that can significantly improve clinical outcomes.

## Data Availability

Not applicable.

## Glossary of terms

AAC: Accelerated Access Collaborative
AAP: Accelerated Access Pathway
ABPI: Association of the British Pharmaceutical Industry
BIA: BioIndustry Association
CDF: Cancer Drugs Fund
Chief investigator (CI): (a) in relation to a clinical trial conducted at a single trial site, the investigator for that site, or (b) in relation to a clinical trial conducted at more than one trial site, the authorised health care professional, whether or not he is an investigator at any particular site, who takes primary responsibility for the conduct of the trial.
CID: Complex Innovative Design
CPRD: Clinical Practice Research Datalink
CRO: Contract Research Organisation
CRUK: Cancer Research UK
CTA: Clinical Trial Authorisation
CTU: Clinical Trials Unit
DHSC: Department of Health and Social Care
ECMC: Experimental Cancer Medicine Centre
EMA: European Medicines Agency
EUNetHTA: European Network for Health Technology Assessment
FDA: US Food and Drug Administration
Funder: The body or bodies that fund a research project.
GCP: Good Clinical Practice
HPRA: Health Products Regulatory Authority
HRA: Health Research Authority
HTA: Health Technology Assessment
ICPV: Independent Cancer Patients’ Voices
IMP: Investigational Medicinal Product
Investigational research / clinical trial: any investigation in human subjects intended to discover or verify the clinical, pharmacological and/or other pharmacodynamic effects of one or more investigational medicinal product(s), and/or to identify any adverse reactions to one or more investigational medicinal product(s) and/or to study absorption, distribution, metabolism and excretion of one or more investigational medicinal product(s) with the object of ascertaining its (their) safety and/or efficacy.
IRAS: Integrated Research Application System
MAMS: Multi-arm, multi-stage
MHRA: Medicines and Healthcare products Regulatory Agency
NCSLC: Non-small cell lung cancer
NHS: National Health Service
NICE: National Institute for Health and Care Excellence
NIHR: National Institute for Health Research
Oversight committee: Having responsibility for the scientific management of the trial.
PD: Pharmacodynamics
PK: Pharmacokinetics
Patients and service users: Recipients of health care, social care or other services or support provided by or on behalf of health or social care organisations, such as NHS patients and social care service users. Includes people receiving integrated health and social care, e.g. Health and Social Care (HSC) users in Northern Ireland. Excludes children’s social care service users in England and Scotland.
PPI: Patient and Public Involvement
Principal Investigator (PI): The lead investigator who is responsible for the local conduct of the trial at a site and to whom other sub-investigators and research team members are accountable.
PROM: Patient Reported Outcome Measure
The public: The general public. Includes carers, relatives of patients and service users and healthy volunteers.
Public involvement: Working in collaboration with patients, service users or the public in the design, management, conduct or dissemination of research.
RCT: Randomised Controlled Trial
REC: Research Ethics Committee
Research site: The organisation with day-to-day responsibility for the location where a research project is carried out.
Research team: The people involved in the conduct of a research project. There may be different research teams for the project at different sites.
SDV: Source Data Verification
SMC: Scottish Medicine Consortium
Sponsor: The person or body who takes on ultimate responsibility for the initiation, management and financing (or arranging the financing) of a clinical trial.
TMG: Trial Management Group

## References

[1] International Agency for Research on Cancer. All cancers fact sheet. Globocan, 2018. http://gco.iarc.fr/today/data/factsheets/cancers/39-All-cancers-fact-sheet.pdf (2018).

[2] International Agency for Research on Cancer. Cancer tomorrow—Estimated number of incident cases from 2018 to 2040, all cancers, both sexes, all ages. https://gco.iarc.fr/tomorrow/graphic-isotype?type=0&;population=900&mode=population&sex=0&cancer=39&age_group=value&apc_male=0&apc_female=0 (2018).

[3] The Institute of Cancer Research. From Patent to Patient—Analysing access to innovative cancer drugs. https://d1ijoxngr27nfi.cloudfront.net/docs/default-source/default-document-library/from-patent-to-patient.pdf?sfvrsn=8fa95f69_2 (2018).

[4] Bhatt, D. L. & Mehta C. Adaptive designs for clinical trials. 10.1056/NEJMra1510061. https://www.nejm.org/doi/10.1056/NEJMra1510061 (2016). 10.1056/NEJMra1510061 27406349

[5] Renfro LA, Mandrekar SJ (2018). Definitions and statistical properties of master protocols for personalized medicine in oncology. J. Biopharm. Stat..

[6] Woodcock J, LaVange LM (2017). Master protocols to study multiple therapies, multiple diseases, or both. N Engl. J. Med..

[7] Antonijevic, Z. & Beckman, R. A. Platform trial designs in drug development: umbrella trials and basket trials. 304 p. https://www.crcpress.com/Platform-Trial-Designs-in-Drug-Development-Umbrella-Trials-and-Basket-Trials/Antonijevic-Beckman/p/book/9781138052451 (2018).

[8] European Medicines Agency. Reflection paper on methodological issues in confirmatory clinical trials planned with an adaptive design. https://www.ema.europa.eu/en/documents/scientific-guideline/reflection-paper-methodological-issues-confirmatory-clinical-trials-planned-adaptive-design_en.pdf (2007).

[9] European Medicines Agency. Reflection paper on risk based quality management in clinical trials. https://www.ema.europa.eu/en/documents/scientific-guideline/reflection-paper-risk-based-quality-management-clinical-trials_en.pdf (2013).

[10] Office for Life Sciences. Life Sciences Industrial Strategy—A report to the Government from the life sciences sector. https://www.gov.uk/government/uploads/system/uploads/attachment_data/file/650447/LifeSciencesIndustrialStrategy_acc2.pdf (2017).

[11] HM Government. Industrial Strategy: Life Sciences Sector Deal 2. https://www.gov.uk/government/publications/life-sciences-sector-deal/life-sciences-sector-deal-2-2018 (2018).

[12] Food and Drug Administration. Master Protocols: Efficient Clinical Trial Design Strategies to Expedite Development of Oncology Drugs and Biologics. Guidance for Industry. Federal Register. https://www.federalregister.gov/documents/2018/10/01/2018-21313/master-protocols-efficient-clinical-trial-design-strategies-to-expedite-development-of-oncology (2018).

[13] Food and Drug Administration. Complex Innovative Designs Pilot Meeting Program. Federal Register. https://www.federalregister.gov/documents/2018/08/30/2018-18801/complex-innovative-designs-pilot-meeting-program (2018).

[14] Shaw, A. T., Riely, G. J., Bang, Y. J., Kim, D. W., Camidge, D. R., Solomon, B. J. et al. Crizotinib in ROS1-rearranged advanced non-small-cell lung cancer (NSCLC): updated results, including overall survival, from PROFILE 1001. Ann Oncol. **30**, 1121–1126 (2019). 10.1093/annonc/mdz131 PMC6637370 30980071

[15] Soda M, Choi YL, Enomoto M, Takada S, Yamashita Y, Ishikawa S (2007). Identification of the transforming EML4-ALK fusion gene in non-small-cell lung cancer. Nature..

[16] Blackhall F, Cappuzzo F (2016). Crizotinib: from discovery to accelerated development to front-line treatment. Ann Oncol..

[17] ECMC. https://www.ecmcnetwork.org.uk/ (2019).

[18] NHS Health Research Authority. Protocol. https://www.hra.nhs.uk/planning-and-improving-research/research-planning/protocol/ (2018).

[19] Agrafiotis DK, Lobanov VS, Farnum MA, Yang E, Ciervo J, Walega M (2018). Risk-based monitoring of clinical trials: an integrative approach. Clin. Ther..

[20] NHS Health Research Authority. HRA and MHRA publish joint statement on seeking and documenting consent using electronic methods (eConsent). https://www.hra.nhs.uk/about-us/news-updates/hra-and-mhra-publish-joint-statement-seeking-and-documenting-consent-using-electronic-methods-econsent/ (2018).

[21] Middleton G, Crack LR, Popat S, Swanton C, Hollingsworth SJ, Buller R (2015). The national lung matrix trial: translating the biology of stratification in advanced non-small-cell lung cancer. Ann. Oncol..

[22] 21st Century Cures Act. https://www.fda.gov/regulatoryinformation/lawsenforcedbyfda/significantamendmentstothefdcact/21stcenturycuresact/default.htm (2019).

[23] European Medicines Agency. PRIME: priority medicines [Internet]. European Medicines Agency—Commission. https://www.ema.europa.eu/en/human-regulatory/research-development/prime-priority-medicines (2016).

[24] HM Government. Accelerated Access Review: Final Report. https://assets.publishing.service.gov.uk/government/uploads/system/uploads/attachment_data/file/565072/AAR_final.pdf (2016).

[25] NHS England. The Accelerated Access Collaborative. https://www.england.nhs.uk/ourwork/innovation/accel-access/ (2019).

[26] Gill, J. & Prasad, V. A reality check of the accelerated approval of immune-checkpoint inhibitors. *Nat. Rev. Clin. Oncol.* **16**, 656–658 (2019). 10.1038/s41571-019-0260-y 31383994

[27] Gyawali B, Kesselheim AS (2018). Reinforcing the social compromise of accelerated approval. Nat. Rev. Clin. Oncol..

[28] National Institute for Health Research. Clinical Trials Toolkit Routemap. http://www.ct-toolkit.ac.uk/routemap/ (2019).

[29] Sydes MR, Parmar MKB, James ND, Clarke NW, Dearnaley DP, Mason MD (2009). Issues in applying multi-arm multi-stage methodology to a clinical trial in prostate cancer: the MRC STAMPEDE trial. Trials..

[30] Barker AD, Sigman CC, Kelloff GJ, Hylton NM, Berry DA, Esserman LJ (2009). I-SPY 2: an adaptive breast cancer trial design in the setting of neoadjuvant chemotherapy. Clin. Pharmacol. Ther..

[31] Liu, S. & Lee, J. J. An overview of the design and conduct of the BATTLE trials. *Chinese Clin. Oncol.* http://cco.amegroups.com/article/view/6846 (2015). 10.3978/j.issn.2304-3865.2015.06.07 26408300

[32] Kaplan R, Maughan T, Crook A, Fisher D, Wilson R, Brown L (2013). Evaluating many treatments and biomarkers in oncology: a new design. JCO..

[33] Harris L, Chen A, O’Dwyer P, Flaherty K, Hamilton S, McShane L (2018). Abstract B080: update on the NCI-molecular analysis for therapy choice (NCI-MATCH/EAY131) precision medicine trial. Mol. Cancer Ther..

